# MiRNA-3978 regulates peritoneal gastric cancer metastasis by targeting legumain

**DOI:** 10.18632/oncotarget.12917

**Published:** 2016-10-26

**Authors:** Yi Zhang, Yuan-yu Wu, Jun-nan Jiang, Xue-song Liu, Fu-jian Ji, Xue-dong Fang

**Affiliations:** ^1^ Department of General Surgery, China-Japan Union Hospital, Jilin University, Changchun, Jilin 130012, China

**Keywords:** gastric cancer, miRNA-3978, legumain, metastasis

## Abstract

Gastric cancer incidence and mortality are among the highest in China, with majority of the mortality related to peritoneal metastasis of gastric cancer. Treatment is limited to radical resection, which is impeded by incidence of metastasis at time of initial diagnosis, thus making it imperative to identify diagnostic and prognostic biomarkers. Legumain, a lysosomal cysteine endopeptidase of the asparaginyl endopeptidase family, has been shown to be overexpressed in patients with metastatic gastric cancer disease and its expression was positively correlated to both disease progression and outcome. However, the mechanism of legumain expression is currently unknown. Legumain overexpression was found to occur at the level of post transcriptional gene regulation. *In situ* prediction algorithms identified legumain as a putative target of miR-3978. MiR-3978 was significantly decreased in peritoneal metastatic tissue specimens and in MKN45 cells that mimic peritoneal metastasis features. Reporter assays using *LGMN* (encoding legumain) 3′ untranslated region (UTR) showed that miR-3978 interacted with the wild-type but not miR-3978-seed mutant. Ectopic expression of miR-3978 mimic in the MKN45 cell line significantly decreased proliferation and suppressed *in vitro* migration and invasion. The miR-3978 mimic inhibited gastric carcinoma and metastatic progression in a mice model by regulating legumain protein expression. Inverse correlation of *LGMN* mRNA and miR-3978 levels in 20 gastric patients at different stages of metastatic disease confirmed the same. Cumulatively, our results indicate that loss of miR-3978 leads to increased expression of legumain, which indicates that miR-3978might be a biomarker for peritoneal metastasis in patients with gastric cancer.

## INTRODUCTION

The Republic of China has one of the highest incidences of gastric cancer globally, with 300,000 patients with gastric cancer projected to die annually [1]. Even though the treatment of choice in gastric cancer is radical resection, 50% of the patients develop peritoneal metastasis [2–7] The major limitation in treating gastric cancer patients with peritoneal metastasis is lack of molecular markers that would lead to early diagnosis, in turn increasing the efficacy of radical resection as a treatment modality, and lack of mechanistic understanding of the processes that drives peritoneal metastasis [8].

It has been shown that the lysosomal cysteine endopeptidase of the asparaginyl endopeptidase family, legumain or asparaginyl endopeptidase (AEP) [9, 10], is overexpressed in patients with metastatic gastric cancer [11, 12]. It has also been shown that legumain facilitates epithelial to mesenchymal transition (EMT) and metastatic progression in gastric cancer through activation of MAPK and Akt signaling pathways and overexpressed in different cancers [13–19]. However, the precise mechanism about what induces legumain expression during metastatic progression of gastric cancer is not known, which was the objective of the present study. Our findings cumulatively indicate that the microRNA-3978 (miR-3978) regulates legumain expression in normal peritoneum. However, during gastric cancer progression miR-3978 expression is suppressed resulting in concomitant increase in legumain expression.

## RESULTS

**Figure 1:**
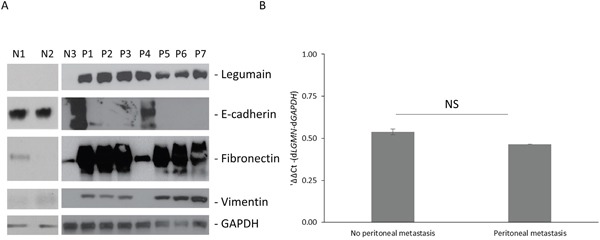
Legumain is overexpressed in gastric cancer patients with peritoneal metastasis **A.** Western blot analysis of legumain expression and indicated epithelial (E-cadherin) and mesenchymal cell (fibronectin and vimentin) markers in samples obtained from normal peritoneum (N) or patients with metastatic gastric cancer (P1-P7). Blots were probed with GAPDH antibody to validate equivalent loading. **B.** Real-time PCR analysis of *LGMN* (encoding legumain) expression in gastric cancer patients with and without peritoneal metastasis (NS: P>0.05).

To explore the mechanism(s) regulating legumain expression, we first evaluated legumain expression in peritoneal metastatic samples obtained from gastric cancer patients. As shown in Figure 1A, legumain was significantly overexpressed in all patient samples tested compared to normal peritoneal wash. Since, legumain was earlier shown to aid in metastatic progression of legumain by inducing EMT (13), we assayed the samples for expression of epithelial cell marker, E-cadherin, and mesenchymal cell markers, fibronectin and vimentin. E-cadherin was detected in only one patient sample, where the mesenchymal markers were also significantly less than the other samples (Figure 1A). Overall, the patient derived samples had correlative expression of legumain and mesenchymal cell markers. To understand if the difference in legumain expression was due to differential transcription of *LGMN* in patients with peritoneal metastasis, we performed qRT-PCR (Figure 1B). There was no significant difference in *LGMN* (encoding legumain) in samples derived from patients with and without peritoneal metastasis, indicating a post-transcriptional regulation of *LGMN* expression.

**Figure 2:**
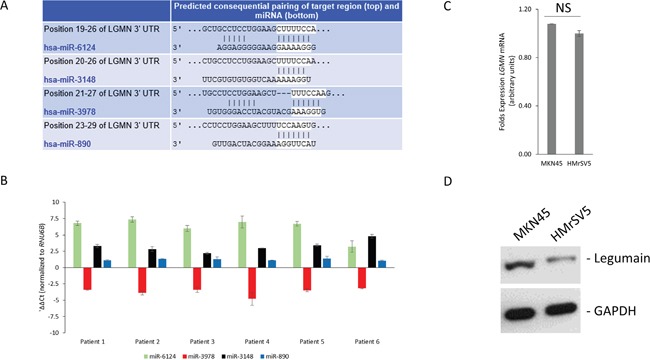
Prediction of *LGMN* as a target of miR-3978 **A.** Complementary seed match between indicated miRNAs and the 3′ UTR of *LGMN* as predicted by TargetScan software. The full complement of predicted miRNAs are indicated in Supplementary Table S1. **B.** Real-time PCR analysis of indicated miRNA expression in gastric cancer patients with peritoneal metastasis (normalized to *RNU6B* and normal peritoneal tissue). **C** and **D.** Real-time PCR (NS: P>0.05) (C) and western blot (D) analysis of legumain expression in indicated cell lines.

To explore the possibility of miRNA-mediated regulation of *LGMN* expression, we used two independent algorithms to *in situ* predict the miRNAs targeting *LGMN*, TargetScan and microCosm (Figure 2A, and Supplementary Table S1). No conserved miRNAs were detected; however, both algorithms detected miR-1624, miR-3148, miR-3978, and miR-890 as putative poorly conserved miRNAs targeting *LGMN*. There was no previous knowledge available of *LGMN* being targeted by miRNAs, hence we decided to pursue it further.

We next determined expression levels of the aforementioned four miRNAs in tumor tissue specimens obtained from gastric cancer patients. As shown in Figure 2B, miR-3978 was the only miRNA that showed significantly suppressed expression in gastric cancer patients with peritoneal metastasis (P<0.05), whereas the other three were either overexpressed or did not show significant change.

To determine if *LGMN* is actually being targeted by miR-3978, we decided of using the well-differentiated cell line mimicking peritoneal metastasis, MKN45, and the human mesothelial cell line, HMrSV5. Even though *LGMN* transcript expression was comparable in these two cell lines (Figure 2C), legumain was significantly overexpressed in MKN45 cells compared to HMrSV5 (Figure 2D). In contrast, miR-2978 expression was significantly higher in the HMrSV5 cells compared to the MKN45 cells (data not shown). Hence, MKN45 and HMrSV5 provided us a good *in vitro* model system to further query the role of miR-3978 in regulation of legumain protein expression.

**Figure 3:**
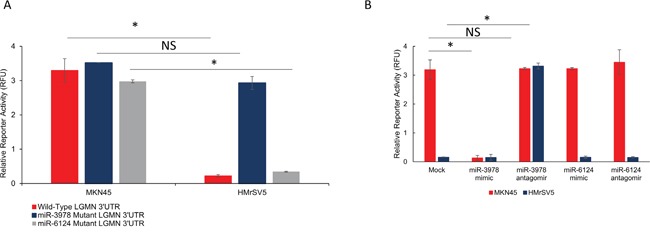
*LGMN* is a bona-fide target of miR-3978 in gastric cancer **A.** Relative luciferase activity of transiently transfected luciferase reporter constructs containing either wild-type or mutated (miR-3978 binding site deleted) *LGMN* 3′ UTR in MKN45 and HMrSV5 cells. **B.** Relative luciferase activity of transiently transfected luciferase reporter constructs containing wild-type *LGMN* 3′ UTR in MKN45 and HMrSV5 cells, either mock transfected or transfected with miR-3978 antagomir and mimic as indicated (NS: P>0.05; *P<0.05).

We next determined if *LGMN* is a bona fide target of miR-3978. To test putative interaction between the 3′UTR of *LGMN* and miR-3978, luciferase reporter constructs containing the wild-type *LGMN* 3′UTR were transfected in MKN45 and HMrSV5 cells. *LGMN* 3′UTR containing reporter were inhibited 3.2 ± 0.05 folds (P = 0.004) in HMrSV5 cells compared to MKN45 cells (Figure 3A). To confirm that the effects observed was due to miR-3978 targeting the *LGMN* 3′UTR, we generated and tested miR-3978 and miR-6124 binding mutants of the *LGMN* 3′UTR reporter (nucleotides 19-26 corresponding to the miR-6124 binding site, and 21-27 corresponding to miR-3978 binding site). The miR-3978 binding mutant *LGMN* reporter did not show any difference in relative luciferase activity between MKN45 and HMrSV5 cells (p>0.05) (Figure 3A), confirming that *LGMN* mRNA was being targeted by the miR-3978 in these cells. However, the miR-6124 binding mutant *LGMN* reporter was still being repressed in the HMrSV5 cells, indicating that *LGMN* is not a bona fide target of miR_6124 (Figure 3A). To further confirm that differential miR-3978, but not miR-6124, expression can modulate the expression of the *LGMN* reporters, the cells were co-transfected with miR-3978 or miR-6124 mimic or antagomir. Whereas miR-3978 antagomir de-repressed *LGMN* 3′-UTR reporter expression in HMrSV-5 cells, miR-3978 mimic abrogated reporter expression in the MKN45 cells (Figure 3B), confirming *LGMN* as a bona fide target of miR-3978 in these cell lines. However, neither miR-6124 antagomir nor mimic resulted in any change of *LGMN* reporter expression in either HMrSV5 or MKN45 cells (Figure 3B), confirming that *LGMN* is not a bona fide target of miR-6124.

**Figure 4:**
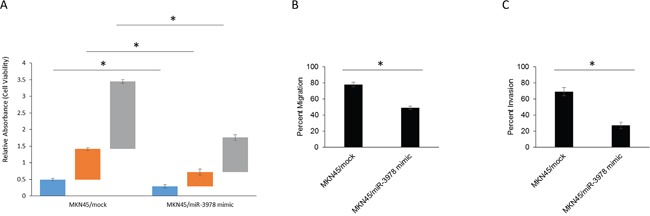
*MiR-3978* expression levels dictate cell viability, migration and invasion abilities in GBM cells **A.** Cell viability was measured in MKN45 cells mock transfected or transfected with miR-3978 mimic at 24, 48, and 72 hours after transfection by the MTT assay. **B** and **C.** Modulation of miR-3978 changes cell migration and invasion abilities in the MKN45 cells. The migrated and invasive cells were photographed using a microscope (data not shown), and the number of the migrated and invasive cells in every field was counted and represented as percent of total cells at the beginning of the assay. Error bars, S.D. *P<0.05.

To determine whether differential miR-3978 expression affects cell proliferation, cell viability assays were carried out with MKN45 cells, mock transfected or transfected with miR-3978 mimic. Transfection with miR-3978 mimic suppressed cell proliferation after day 1 (mock vs miR-3978 mimic – 0.49 ± 0.04 vs 0.29 ± 0.05, p<0.05), day 2 (mock vs miR-3978 mimic – 0.92 ± 0.04 vs 0.43 ± 0.09, p<0.05), and day 3 (mock vs miR-3978 mimic – 2.04 ± 0.06 vs 1.04 ± 0.09, p<0.05) (Figure 4A). Our results suggested that legumain potentiates cell proliferation in metastatic gastric cancer that can be inhibited by miR-3978 expression.

We then scored each of the individual transfectants MKN45 cells (mock and miR-3978 mimic), for migration (Figure 4B) and invasion (Figure 4C) in standard transwell assays. Using these criteria, phase contrast imaging (data not shown) and quantification showed that overexpression of miR-3978 inhibited *in vitro* migration (mock vs miR-3978 mimic – 78 ± 3 vs 49 ± 2, p<0.05) (Figure 4B), and invasion (mock vs miR-3978 mimic – 69 ± 5 vs 27 ± 4, p<0.05) (Figure 4C). Our results suggested that miR-3978 expression can inhibit *in vitro* migration and invasion in gastric cancer, which occurs by repression of *LGMN* expression. Cumulatively, the profound repression in relative expression of miR-3978 and increased expression of legumain in metastatic gastric cancer tissue or cell line along with its capacity to impinge *in vitro* migration and invasion suggested that it may drive tumorigenesis and metastatic progression in gastric cancer.

**Figure 5:**
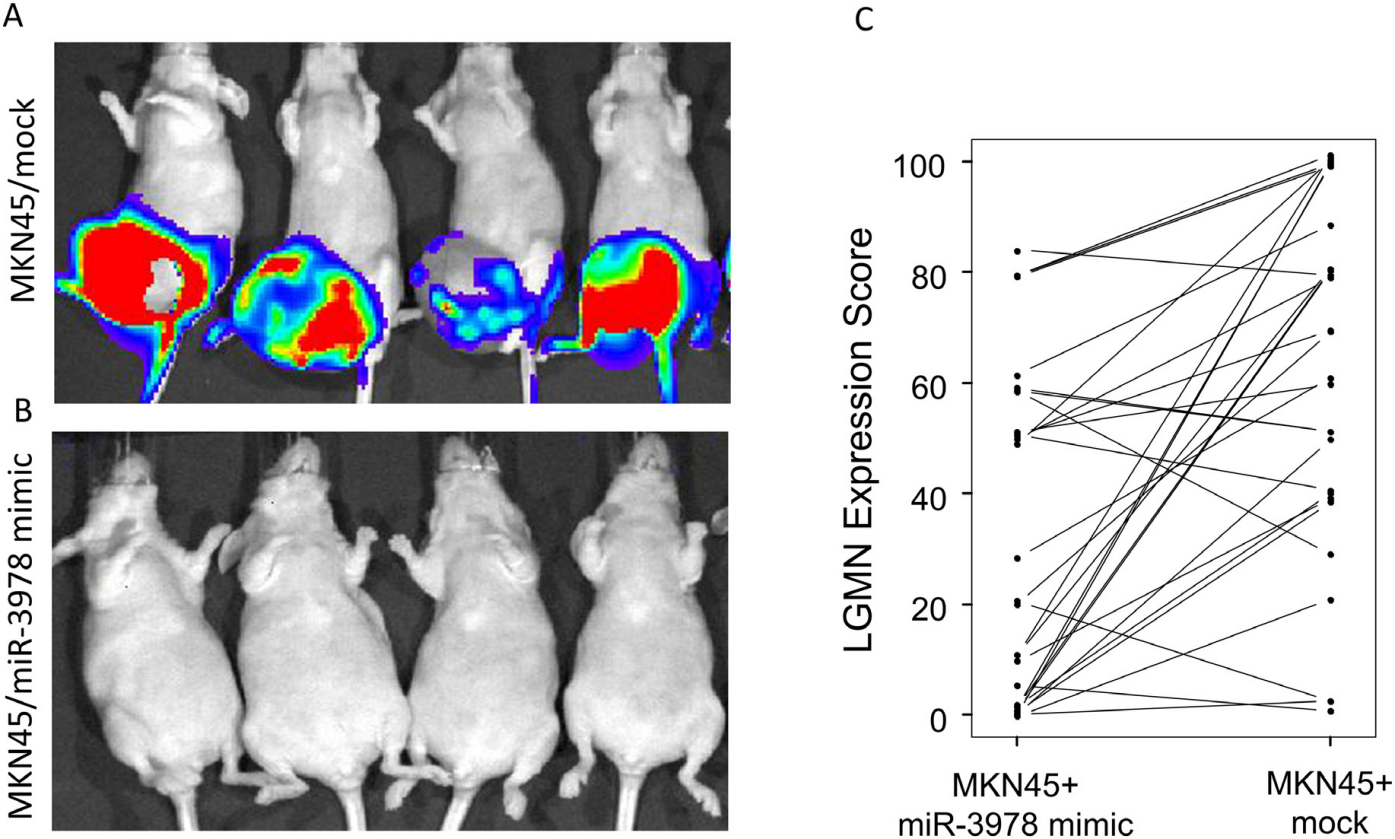
MiR-3978 expression levels dictate peritoneal metastasis of gastric cancer **A** and **B.** The effect of miR-3978 overexpression on metastasis was examined. MKN45 cells stably overexpressing either scrambled control or mR-3978 mimic were used. The incidence of tumor formation and metastasis were measured by luciferin injection and bioluminescence imaging of Firefly Luciferase. **C.** Relative legumain protein expression as assessed by the percent score in the 29 animals in the two experimental groups.

Having shown that miR-3978 suppressed tumor cell growth in MKN45 cells, we investigated its antitumor effects in a murine model of gastric cancer. Ectopic expression of miR-3978 in MKN45 cells ameliorated tumor growth as compared to mock transfected MKN45 cells (Figure 5A and 5B). When tissue samples obtained from each of 29 animals in this two experimental groups were assayed for legumain expression, we noticed a significantly higher legumain expression in animals that were not transfected with MKN45 cells (P<0.05) (Figure 5C). These data suggest that miR-3978 expression is inversely correlated to legumain expression and high expression of miR-3978 can potentially suppress gastric cancer formation and progression.

**Figure 6:**
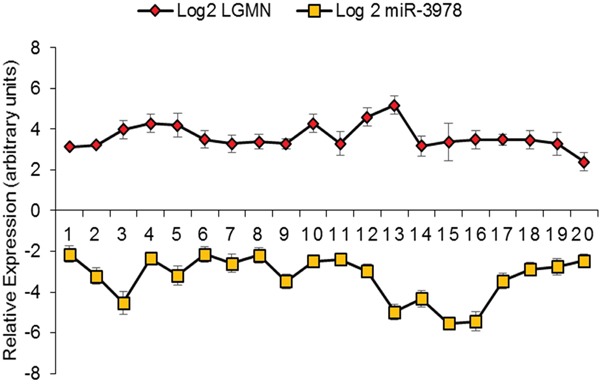
MiR-3978 and legumain levels are inversely correlated in gastric cancer patients with metastatic disease Pearson correlation demonstrating the inverse relation between miR-3978 (determined by qRT-PCR) and legumain (determined by immunohistochemistry) in paired samples (*p* < 0.05, Pearson correlation r = −0.8761).

Given that our experiments indicated that legumain is a bona-fide target of miR-3972, we hypothesized that suppression of miR-3978 expression might be an underlying feature of peritoneal metastasis pathogenesis in patients with gastric cancer. We determined miR-3978 and legumain protein expression in 20 gastric cancer patients with different stages of peritoneal metastasis. Our results indicated a dynamic and inverse correlation between down-regulation in the levels of miR-3978 and the observed increase in legumain protein expression in metastatic gastric cancer tissue specimens (Figure 6) (P <.005, Pearson correlation r = −0.8761).

## DISCUSSION

In the current study, our experimental results show that miRNA-3978 and legumain is downregulated and upregulated, respectively, in gastric cancer patients with peritoneal metastasis. Repression of legumain expression by using miRNA-3978 mimic inhibited cell proliferation, as well as migration and invasion. Finally, modulating miR-3978 expression levels directly correlated to metastatic potential of the MKN45 cells in xenograft experiments. Cumulatively, this highlights loss of miR-3978 expression as potential prognostic biomarker in gastric cancer patients. It will be interesting to investigate in future research endeavors how miR-3978 and legumain expression varies and correlates to disease progression in patients that had either radiation therapy or adjuvant chemotherapy post-radical resection of the primary tumor. It would also be interesting to determine additional targets of miR-3978. To the best of our knowledge, putative targets of miR-3978 have not been defined until this study.

MiRNAs are evolutionarily conserved 21-23 nucleotides RNAs that regulate post-transcriptional gene expression either by blocking translation or degrading target messenger RNAs (mRNAs) [21, 22]. Given that our data showed that miR-3978 targeted *LGMN* transcript, but there was no overarching decrease in steady state expression of *LGMN* mRNA in patients with peritoneal metastasis, it can be envisioned that miR-3978 does not degrade *LGMN* transcript, but instead inhibits its translation. In fact, our *in vitro* reporter assays corroborate this hypothesis. It will be important in future to define the precise translational inhibition mechanism by which miR-3978 inhibits *LGMN* translation. MiRNAs can function in both normal and transformed cells, thus functioning as tumor suppressors or oncogenes, and have even been shown to play a role in metastasis [23–26].

Not much is known about role of miR-3978 in gastric cancer or in normal physiology. There has been one report that suggested differential expression of miR-3978 in lung cancer patients [27]. Given that legumain is overexpressed in different tumors, it will be interesting whether loss of miR-3978 expression is a pervasive mechanism.

It will also be of potential interest to study how miR-3978 is regulated and what causes its suppression during metastatic progression of gastric cancer patients. Conversely, it will be important to verify if other known or predicted targets of miR-3978 are differentially expressed in gastric cancer patients with metastatic disease and whether they functionally contribute to the pathogenesis of peritoneal metastasis. Finally, peritoneal metastasis can happen as a result of colorectal or hepatic carcinoma too; hence, it will be imperative to correlate miR-3978 expression with stage of gastric and other cancers with potential of peritoneal metastasis. A complete understanding of miR-3978 mediated regulation of legumain might emerge from experiments looking at gene expression following overexpression and knockdown of miR-3978 in *in vitro* platforms

## MATERIALS AND METHODS

### Patients and tissue samples

The study protocol was approved by the Institutional Review Board of Sino-Japanese Friendship Hospital. A total of 20 patients (12 men and 8 women) who had surgery for gastric cancer between 2014 and 2015 at Sino-Japanese Friendship Hospital were included in this study after providing signed informed consent. The mean age of the patients was 61.34 years (range, 39-78 years). The inclusion criterion was presence of peritoneal metastasis at the time of initial presentation as confirmed by two independent pathologists. Paired tumor tissue and normal gastric tissue specimens were obtained from all patients included in the study.

### Cell culture

MKN45 and HMrSV5 cell lines were purchased from the cell bank of Chinese Academy of Sciences. Cells were maintained in RPMI1640 medium (Life Technologies, Shanghai, China) supplemented with 20% FBS (Lonza, Germany). All cells were cultured in a 5% CO_2_ humidified atmosphere and at 37°C.

### RNA, miRNA extraction and qRT-PCR

RNA isolation and qRT-PCR to query *LGMN* expression was done as described previously (12). MiRNA from cells and tissues were extracted by the mirVana miRNA isolation kit (Life Technologies, Shanghai, China) according to the manufacturer's instructions. The expression levels of indicated miRNAs and *RNU6B* were detected by TaqMan miRNA assays (Life technologies, Shanghai, China). The −ΔΔCt method was used to analyze the data in each case and normalization was done to *GAPDH* and *RNU6B* expression for mRNA and miRNA, respectively.

### Cloning, transfection, and luciferase assays

The *LGMN* 3′ UTR clone in pMirTarget was obtained from Origene. The *LGMN* 3′ UTR reporter was constructed by amplifying the endogenous *LGMN* 3′ UTR from the Origene. XhoI and ApaI sites were added to the 5′ and 3′ ends of the fragment during the preceding PCR reaction and cloned into the XhoI and ApaI site on the Rr-luc-6XCXCR4 Renilla luciferase vector (Addgene). To make the *LGMN* 3′UTR mutant construct, site-directed mutagenesis was used to delete nucleotides 19-26 or 21-27, corresponding to the miR-6124 and miR-3978 binding sites, respectively. A firefly luciferase vector was used as transfection and normalization control in all luciferase assays. Constructs were sequence verified before being used in experiments. Indicated cells (4×10^4^) were transiently transfected using Lipofectamine 3000 (Life Technologies, Shanghai, China) as per the manufacturer's instructions. Where indicated, cells were transfected with miR-3978 or miR-6124 mimic or antagomir (Life Technologies, Shanghai, China) along with the *LGMN* 3′UTR reporters. Ninety-six hours after transfection, the renilla and firefly luciferase activities were measured consecutively using Dual-luciferase reporter assay system (Promega, Madison, Wisconsin, USA) as per manufacturer's protocol. Each reporter plasmid was transfected at least twice in triplicate. Post-normalization the data was represented as relative fluorescence units (RFU) ± standard deviation (SD).

### Cell proliferation assay

The MTT assay kit (Sigma-Aldrich, St. Louis, MO, USA) was used to measure cell proliferation rates. The relative optical density (OD), as mean ± standard deviation was calculated.

### *In vitro* transwell migration and invasion assays

Culturex 96 well BME cell invasion and cell migration kits (R&D Systems, Marlborough, MA, US) were used to measure *in vitro* migration and invasion, respectively. Percent migration and invasion were calculated and expressed as mean ± standard deviation.

### Animals

Six week-old male BALB/c nude mice under SPF conditions were obtained from Vital River Laboratories (Beijing, China) (approved by the Institutional Review Board of Sino-Japanese Friendship Hospital). The tumor model was done as described previously (20) using parental MKN45 cells or MKN45 cells expressing miR-3978 mimic (n=29 in each group). The cells were stably transfected with firefly luciferase before use in animal experiments to aid in *in vivo* luciferase imaging to detect progression of tumor formation and metastasis. Tissue microarrays (TMAs) were made from paired tumor and non-tumor tissue in each experimental group for comparison of legumain expression.

### Immunohistochemistry

Immunohistochemistry was performed to determine legumain expression levels in patient samples as well as those obtained from the *in vivo* xenograft experiments. Tissue specimens were stained with anti-legumain antibody (Santa Cruz, 1:200 dilutions) and subsequently scored as described previously (12). Scoring was independently performed by two pathologists blinded to the identity of the tissue specimens.

### Statistical analysis

All analyses were done by using the SPSS statistical software program version 18.0 (IBM Corporation, NY). Quantitative variables are presented as the mean ± standard deviation. Data analysis was performed using one-way ANOVA. Tukey's post-hoc test was used to determine the statistical significance in all pairwise comparisons of interest. Pearson correlation coefficients were computed to assess the association between ordinal miR-3978 and legumain. P<0.05 was considered to represent a statistically significant difference.

## SUPPLEMENTARY TABLE





## Abbreviations

UTR: untranslated region
EMT: epithelial to mesenchymal transition
AEP: asparaginyl endopeptidase
miR-3978: microRNA-3978
LGMN: legumain
mRNAs: messenger RNAs
