# Supplementary material for: MiRNA-3978 regulates peritoneal gastric cancer metastasis by targeting legumain
Source: Oncotarget. 2016 Oct 26;7(50):83223–30. doi: 10.18632/oncotarget.12917 (PMC5347764; doi:10.18632/oncotarget.12917)
Supplement: Supplementary file 1 [file oncotarget-07-83223-s001.pdf]

MiRNA-3978 regulates peritoneal gastric cancer metastasis by targeting legumain

SUPPLEMENTARY TABLE

Supplementary Table S1: TargetScanHuman (Release 7.1) based prediction of putative miRNAs binding to the 3'UTR of *Homo sapiens LGMN*. Context++ score and features that contribute to the context++ score are evaluated as in Agarwal et al., 2015. Conserved branch lengths and PCT are evaluated as in Friedman et al., 2008, with an expanded 84-species alignment as described in Agarwal et al., 2015. No conserved sites were detected.

See Supplementary File 1
